# Disclosure of Alzheimer’s disease blood-based biomarker results in a primary care setting: Opportunities and challenges

**DOI:** 10.1016/j.tjpad.2025.100310

**Published:** 2025-07-25

**Authors:** Corey J. Bolton, Ayda Rostamzadeh, Nathaniel Chin, Nicole R. Fowler, Judith Heidebrink, Annalise Rahman-Fillipiak, Raymond R. Romano, Lindsay R. Clark

**Affiliations:** a Vanderbilt Memory and Alzheimer’s Center, Vanderbilt University Medical Center, Nashville, TN 37203, USA; b Department of Medicine, Vanderbilt University Medical Center, Nashville, TN 37232, USA; c Department of Psychiatry, University of Cologne, Medical Faculty, Cologne 50931, Germany; d Wisconsin Alzheimer's Disease Research Center, School of Medicine and Public Health, University of Wisconsin - Madison, Madison, WI 53792, USA; e Department of Medicine, Division of Geriatrics & Gerontology, University of Wisconsin School of Medicine and Public Health, University of Wisconsin - Madison, Madison, WI 53792, USA; f Department of Medicine, Indiana University School of Medicine, Indianapolis, IN 46202, USA; g Indiana University Center for Aging Research, Indianapolis, IN 46202, USA; h Department of Neurology, University of Michigan, Ann Arbor, MI 48109, USA; i Michigan Alzheimer's Disease Center, University of Michigan, Ann Arbor, MI 48109, USA; j Research Program on Cognition & Neuromodulation Based Interventions, Department of Psychiatry, University of Michigan, Ann Arbor, MI 48109, USA; k Department of Neurology, Vanderbilt University Medical Center, Nashville, TN 37232, USA; l Geriatric Research Education and Clinical Center, William S. Middleton Memorial Veterans Hospital, 53792, USA

**Keywords:** Alzheimer's disease, Blood-based biomarkers, Disclosure, P-tau217, Primary care

## Abstract

Blood-based biomarkers (BBMs) for Alzheimer’s disease (AD) have advanced rapidly and may be a critical tool for broad community-based screening for AD and detection of AD pathology in individuals with cognitive impairment. To meet the impending demand for AD diagnosis, BBMs could be implemented in a primary care setting to maximize accessibility and efficiency. However, this primary care implementation would be associated with numerous challenges, including issues related to disclosure of test results to patients. In this perspective article, we highlight the need for and potential challenges of AD BBM results disclosure in a primary care setting. Drawing from existing studies of AD risk disclosure, we highlight key areas of consideration to maximize patient safety and comprehension of results. Resources are suggested to aid health systems in implementing BBM testing in primary care settings. Finally, we emphasize the need for further research on the accuracy of BBMs and the practice of disclosure in primary care settings.

## 1 Introduction

Alzheimer’s disease (AD) is a complex and prevalent condition that poses a significant challenge to existing healthcare systems [1]. Biomarker confirmation of AD has been limited by costly and invasive methods and has offered limited clinical utility due to a lack of treatment options once a diagnosis is reached. The recent approvals of three anti-amyloid therapies have increased efforts that focused on early identification of symptoms and diagnosis as well as public awareness about detection and treatment. Public awareness and demand for clinical evaluation is at an all-time high yet current diagnostic methods are not scalable to meet this demand [2].

Blood-based biomarkers (BBMs) for AD have emerged in recent years and represent the most promising diagnostic tool for AD at the population level [3]. Plasma assays measuring quantities of phosphorylated tau (p-tau) [4], beta-amyloid (Aβ) [5], neurofilament light (NfL) [6], and glial fibrillary acidic protein (GFAP) [7], amongst others, have shown great promise at identifying AD pathology (i.e., Aβ plaques and tau neurofibrillary tangles) or concomitant processes (e.g., reactive astrocytosis, neurodegeneration) and predicting future cognitive decline. While new BBMs are constantly being developed, at present, a specific isoform of p-tau, namely p-tau217, has shown a high degree of accuracy in detecting AD pathology in the brain [4]. First measured in the blood in 2020 [8], there are now over 100 publications describing the efficacy of p-tau217 at identifying AD pathology [4], differentiating AD from other neurodegenerative diseases [8], and predicting clinical decline [9]. Recent work using a ratio of phosphorylated to non-phosphorylated p-tau217 has shown similar or better performance in detecting amyloid plaques and tau tangles as current FDA-cleared cerebrospinal fluid assays [10]. These studies have advanced the field of AD diagnostics, such that this biomarker is rapidly moving towards widespread clinical implementation, not only in specialty care settings, but also in primary care. While p-tau217 shows great promise as a standalone test, the future of BBM testing in primary care may include a panel of tests including p-tau217 as well as other markers of core AD pathology (e.g., Aβ) and concomitant features (e.g., GFAP, NfL).

With an ever-increasing clinical demand and more potential treatments for AD on the horizon, specialty memory clinics alone are unlikely to be able to meet the demand, and screening for, and diagnosis of, AD will instead occur more frequently in primary care [2]. Already, the vast majority of dementia diagnoses are being given outside of specialty memory clinics [11]. Primary care clinics are the frontline of healthcare delivery and provide unrivaled access to patients, thus situating the primary care clinic as a desirable setting to meet the need of large scale BBM testing. Widespread access to BBM testing could help to mitigate some of the existing health disparities in AD diagnosis. Individuals from underrepresented racial/ethnic communities have higher rates of dementia, but are consistently diagnosed less frequently, later, and less accurately than non-Hispanic Whites [12]. These disparities may be due in part to lack of access to specialized care; equipping primary care clinics with the ability to screen for AD with BBMs could be one step towards addressing these disparities. While the potential benefits of detecting and diagnosing AD in primary care clinics are numerous, there are certainly challenges in implementing this approach. In particular, communicating BBM results to patients in a primary care clinic is complex and will require additional training and resources for PCPs.

The aim of this perspective paper is to bring attention to the unique challenges of disclosing BBM results in a primary care setting and highlight potential resources to mitigate these challenges. This discussion primarily focuses on the primary care model in the United States, although the challenges identified are likely relevant in numerous settings. Additionally, this discussion is intentionally centered on patients with cognitive symptoms, including those presenting with subjective or objective cognitive impairment. However, it should be noted that this discussion is also relevant for population-based screening for AD pathology, which could be a reality should novel treatments receive approval for preclinical administration as a preventive measure. The disclosure of BBMs is the main focus, rather than other relevant AD risk information (e.g., *apolipoprotein-E (APOE)* ε4 carrier status), as BBMs provide the greatest potential for clinical implementation in a primary care setting. Disclosure practices regarding genetic or other diagnostic tests are also worth consideration but given that these tests are more likely to be used in a specialty care clinic, they are not the primary focus of this paper.

The Advisory Group on Risk Evidence Education in Dementia (AGREEDementia) is a multidisciplinary working group focused on informed discussions and development of educational material to support the communication of the results of genetic and biomarker-based prediction, detection, and quantification of dementia risk [13,14]. The symptomatic subcommittee of AGREEDementia specifically focuses on issues related to disclosure of biomarker results to individuals with cognitive or behavioral impairment. Drawing from the collective wealth of knowledge amassed through the study of biomarker disclosure practices in AD research, we hope to offer both practical steps to ensure patient safety as well as aspirational goals for health systems to strive towards when considering patient care in this new era of AD diagnosis and management.

## 2 Primary care clinics are an ideal setting for BBM testing

Incorporation of BBMs by primary care could expedite access to dementia specialists for novel AD therapies. Current estimates based on the availability of dementia care specialists and the population of older adults who might be eligible for treatment suggest that wait times for monoclonal antibody treatments for AD could be up to four years by 2027 [2]. The integration of BBMs alongside cognitive screening may help reduce specialty care wait times by triaging patients and referring only those at highest risk for future decline, and/or those most likely to be eligible for treatments (e.g., positive BBM and abnormal cognitive test results). The addition of BBMs to a traditional cognitive screening measure substantially reduced wait times from 50 months to 12 months in one recent projection [15].

The extended wait times projected for novel therapies are problematic as timely diagnosis and initiation of treatment is essential. Given the presumed early role of amyloid pathology (i.e., Aβ) in the pathophysiological cascade of AD, early initiation of treatment may produce stronger benefits. Subgroup analyses of the recent TRAILBLAZER-ALZ2 study of donanemab support this hypothesis, showing stronger treatment effects in individuals with mild cognitive impairment (MCI; 39.3 % slowing of decline) compared to those with mild AD dementia (19.2 % slowing) [16]. Rate of annual volume loss in the entorhinal cortex of individuals with MCI is estimated to be ∼4 % [17], thus expediting treatment by even months could avoid substantial neuronal loss.

Beyond increasing access to novel anti-amyloid therapies, there are numerous benefits to obtaining an early and accurate diagnosis of AD that can be facilitated by integration of BBMs into primary care clinics. As cognitive decline is often multifactorial, the most effective approaches to treatment are likely multi-pronged [18]. Behavioral and lifestyle interventions are effective means of reducing risk of dementia [19] and can be promoted during AD test disclosure [20], regardless of whether biomarker results are positive or negative [21]. Additionally, biomarker testing in primary care can facilitate further workup to identify causes of cognitive decline in individuals who are AD biomarker negative. If results are positive, continued cognitive decline is more likely and patients will have an opportunity to explore advanced care options and financial implications more effectively. For the PCP, knowing a patient’s biomarker result can aid in making other treatment decisions and inform the need for additional monitoring, aid in medication adherence, or the need for considering a collateral informant’s report on current health status.

Primary care clinics are a pivotal component of healthcare delivery, serving as the frontline of diagnosis and screening efforts for a multitude of conditions. The indispensable role of PCPs is evident in the field of cancer diagnosis and treatment; through regular screenings and proactive monitoring, PCPs identify potential signs and symptoms of cancer at its earliest stages, when treatment options are often most effective. Patients who utilize primary care services more often prior to their cancer diagnosis have markedly better long-term outcomes, including a 39 % reduction in odds of metastatic disease and 21 % reduced risk of cancer-related death, than those not under routine monitoring by their PCP [22]. In addition to providing critical assistance with screening, involvement of PCPs in the care of patients with chronic conditions can extend beyond initial diagnosis to encompass holistic health management and long-term supportive care. PCPs are already managing the comorbidities that put their patients at increased risk of AD, so they are well positioned to help communicate risk for decline and strategies for reducing risk of dementia.

## 3 Interest and reactions to learning biomarker results

Full transparency and fast delivery of health information is a key element of patient-centered care [23]. This principle emphasizes that comprehensive health information is shared in a timely manner with patients to promote informed decision making. In the US, the 21st Century Cures Act has put this idea into law by requiring that test results be made available to patients electronically as soon as possible, with only a few exceptions. This reflects the fact that the vast majority of people (>95 %) want to know their test results right away [24]. While patients favor the immediate release of results, healthcare professionals prefer to delay release to allow time for provider communication rather than unsupervised communication of results [25]. As of 2022, more than half of US adults surveyed reported accessing their health information online, more than twice as many as in 2014 and *a* > 50 % increase from 2020 [26]. The COVID-19 pandemic is likely a large driver of this rapid increase, with patients logging onto their portal to review test results, thus highlighting the potential of electronic health portals for rapid dissemination of sensitive medical test results. While this trend has many positive aspects such as increasing patient ownership of their health, patients often view their test results before the ordering clinician has a chance to review the results [27], thus increasing the likelihood of misinterpretation or confusion.

Even for a much-feared diagnosis such as AD, people desire to know their diagnosis. In a 2013 international survey, two-thirds of older adults reported that they would pursue diagnostic testing for AD if such a test were available, despite no available disease modifying therapies [28]. These findings have been consistently replicated in diverse patient populations. Over 95 % of older adults and their care partners enrolled in research studies on aging and dementia endorsed wanting their biomarker results, with no differences depending on race or demographic factors such as socioeconomic status or access to healthcare [29]. A recent retirement community survey showed that, even amongst cognitively healthy older adults, three-fourths of participants reported that they would want to receive their results of a blood test for AD risk [30]. Nearly three out of four caregivers of patients with cognitive decline also reported a desire to know the results of AD BBM tests, citing actionability, explanation for symptoms, and opportunities for better care and future treatment as reasons for wanting their loved one’s results [31].

Available data suggests that disclosure of AD risk or biomarker test results can be performed safely and have numerous benefits. In research settings, disclosure of AD dementia risk based on *APOE*-ε4 carrier status and amyloid positivity on positron emission tomography (PET) has been shown to be safe [32,33], and may result in some benefit, as participants who learn of their increased risk have 173 % higher odds of making positive health behavior changes including diet and exercise [20] and are about 6x more likely to plan for long-term care [34]. Although patients with MCI and their caregivers in an amyloid PET disclosure-focused randomized trial did not have significant increases in depressive symptoms following a positive amyloid PET scan, moderate and sustained emotional distress was observed [35]. In the Imaging Dementia Evidence for Amyloid Scanning study, care partners of patients with dementia expressed relief and gratitude regarding positive amyloid PET results disclosure, while care partners of patients with MCI had 50 % higher odds of reporting symptoms of anxiety following learning the results of a positive amyloid PET scan [36]. Overall, the literature suggests that AD biomarker testing, while at times accompanied by some understandable distress in the case of a positive result, is not associated with any long-term psychological harm.

There remain many unanswered questions regarding the safe and effective practice of AD BBM disclosure. Perhaps most significantly, there is a dearth of literature investigating the effects of disclosure of blood-based biomarker results or of disclosure of any biomarker results in clinical settings. Research studies on aging and dementia, and especially those specifically investigating disclosure, are prone to significant selection biases that limit the broad applicability of findings to other settings, including primary care. Patients who volunteer to participate in AD biomarker research often do so to learn of their own results [37]; in a clinic setting, patients’ desire to learn their results may be mixed. Typically, patients with severe mental illness and/or substance use disorders are excluded from studies. Primary care BBM implementation would require disclosure to patients who are psychiatrically diverse and may be less knowledgeable about AD. In the absence of evidence for the safety of BBM disclosure in patients with significant psychiatric symptoms, there is a need for pre-test screening for psychiatric symptoms, as well as post-disclosure follow-up to monitor psychological response. Additional visits and screenings are not always covered by insurance and add to the time burden associated with disclosure in fast-paced clinics. More work must be done to better understand the unique challenges and barriers to effective and safe disclosure in clinical settings.

## 4 Challenges associated with the communication of BBM results

The role of the PCP in AD will likely be restricted to screening and diagnosis rather than delivery of newer therapies given the specialized testing, ongoing monitoring, and potential side effects associated with current immunotherapy options. A two-step model has been proposed in which early detection with BBMs is conducted and then further confirmatory testing is sought in cases which are ambiguous [38]. In this model, PCPs would be the ideal administrators of the first step of BBM testing given the significant demand. Although PCPs will likely not have to bear the responsibility of treatment, there are significant challenges associated with even this first stage of BBM testing that should be considered.

Ordering a BBM or panel of BBMs necessarily implies that the PCP will be able to interpret and explain the results to the patient. Even if the patient will be referred to a specialist, given potential wait times and differing desires for pursuing further workup, the ordering physician has a duty to explain test results. AD pathogenesis is complex with shifting prominence of pathologies as the disease progresses. This complexity underscores the significance of biomarker-based diagnostics, yet interpreting these biomarkers presents a formidable challenge, particularly for PCPs lacking specialized training. While the accumulation of amyloid serves as a hallmark early event in AD, the disease's progression into symptomatic stages is associated with the ascendance of other pathologies. Amyloid biomarkers exhibit a plateau in symptomatic disease stages, while biomarkers of late-stage changes, such as neurodegeneration, assume greater predictive significance. There is evidence to suggest that certain BBMs perform differently at different disease stages. Plasma levels of p-tau217 predict cognitive decline [9,39] and amyloid status [4,9] in preclinical AD and MCI, but plasma p-tau levels do not associate with CSF or PET biomarkers in the dementia stage [40]. Plasma NfL levels, on the other hand, predict cognition in individuals with clinical dementia or MCI, but not in preclinical disease stages [41,42]. There is further evidence from the CSF biomarker literature to suggest that fluid biomarkers of AD show differing associations with clinical outcomes when examined across different disease stages. For example, CSF amyloid levels have been associated with cognition in subjective cognitive decline, while CSF p-tau levels were associated with cognition in MCI; neither biomarker was associated with cognitive functioning in dementia [43]. The predictive accuracy of BBMs is improved when simultaneously considering the patient’s cognitive status [44]. If cognitive impairment is not confirmed, positive BBM results could be misinterpreted as overly dire (e.g., if AD changes are actually asymptomatic). Further, there is an increased risk of misdiagnosis in individuals without cognitive impairment due to a greater risk of false positive BBM results when assessed in a population with a low prevalence of AD pathology (i.e., cognitively unimpaired individuals).

While AD pathology is a significant contributor to cognitive impairment, it is crucial to recognize that cognitive decline can stem from various factors beyond AD pathology alone. A positive biomarker result indicating AD pathology, even in the presence of cognitive impairment, does not conclusively attribute AD as the sole or primary cause of the individual's impairment. Instead, biomarker results must be contextualized within the broader framework of the patient's clinical presentation and overall health. Additionally, since BBMs are not 100 % accurate compared to the clinical standard (e.g., amyloid PET scan), PCPs will need to understand the test limitations and factors that can influence accuracy. For example, medical comorbidities such as chronic kidney disease and obesity may alter biomarker levels and confound results [45]. Interpretation of fluid biomarkers in AD is nuanced, demanding a deep understanding of AD pathology, clinical staging, comorbid conditions which affect cognition, and biomarker intricacies to ensure accurate diagnosis and effective communication of results. PCPs who are not trained in the intricacies of AD pathology and biomarkers may find it difficult to interpret results themselves, much less explain results to patients.

In addition to challenges with interpretation of results, communicating these nuanced results to participants with cognitive impairment presents another challenge. This is true in all settings, but perhaps more so in primary care settings where visit times tend to be shorter and memory changes are often not the sole focus of the visit. If results are communicated to the patient only, without a cognitively intact loved one or care partner present, there is an increased likelihood that results will be forgotten or misremembered. Further, disclosing an individual’s risk of dementia or diagnosis of AD is inherently associated with some degree of psychological risk. Thus, when communicating results, there is an impetus to monitor the patient’s and their loved ones’ psychological response to the information so that further assessment and/or referral for mental health services is offered when needed. Given these challenges, the potential for misinterpretation or miscommunication of results in a PCP setting is considerable (see Table 1 for summary). These challenges are particularly worthy of addressing due to the potential for harm to patients and their loved ones.

**Table 1:** Potential challenges in BBM disclosure.

*Misinterpretation*	*Communication Issues*	*Missed Opportunities*
Test is negative and patient is told they do not have AD and no additional work-up for symptoms is completed	Results are forgotten if not communicated to loved one in addition to patient, no written communication of results is provided	Psychological impact of disclosure in some individuals could be missed, these patients may not be plugged in with resources that could be helpful
Test is positive and risk of dementia is overstated (e.g., imminent risk in an individual with no or minimal cognitive impairment)	If EHR allows for patients to see results prior to discussing with their doctor, patients may misinterpret results	Entire treatment plan may revolve around biomarker results and other contributing (or primary) factors could be overlooked
Test is false positive or false negative due to comorbidities or in an under-represented population lacking sufficient validation, etc.		

## 5 Lessons learned in AD biomarker result communication

While the literature surrounding disclosure of BBM results in AD is nascent, there is a substantial body of evidence on the effective disclosure of AD risk (e.g., *APOE* genetic status) or biomarker (e.g., amyloid PET) results. While there are some unique challenges associated with disclosure of BBMs in a primary care setting that limit the generalizability of past research to this area, there are still many lessons that may be gleaned (see Table 2 for summary).

**Table 2:** Lessons learned in AD biomarker disclosure.

*Pre-disclosure*	*During Disclosure*	*Post-disclosure*
Patient education regarding what BBMs do and do not mean	Aiming to communicate quantitative results (as opposed to binary positive or negative)	Monitoring psychological response over time
Education tailored to patient’s current cognitive status	Using specialized communication techniques to promote health literacy	Encouraging active engagement in brain health strategies
Expectation setting regarding potential ambiguity of results	Using teach back methods to ensure understanding	Ensure patient follow through with treatment recommendations
Assessing psychological readiness to receive results	Encouraging patient engagement by eliciting questions frequently	Identify and troubleshoot barriers for patients pursuing treatment

A consistent theme in the study of biomarker result communication is the importance of pre-disclosure counseling and patient education [46]. Given the complexities of BBM interpretation (as discussed above), patients may benefit from an initial discussion regarding what information the test results can and cannot convey. For instance, patients could be made aware that a negative test does not mean that they do not have cognitive impairment nor that they will not develop dementia. Likewise, a positive test result does not mean that AD is the primary cause of their symptoms or that they certainly will develop dementia (in patients with MCI). Setting expectations regarding the ambiguity of results prior to disclosure can improve the patient’s experience of receiving the results. Pre-disclosure education should be tailored to an individual’s cognitive status, as the practical implications of BBM results can be drastically different for individuals who are cognitively unimpaired compared to those with mild cognitive impairment or dementia [47]. Pre-disclosure counseling can also provide an opportunity to assess the patient’s psychological readiness to receive their results. Simple questions such as “How would you feel if your test result was positive?” or “What steps would you take if you found out your test result was positive?” can provide key insights into the safety of disclosing results. Stemming from this education, a process of shared-decision making is essential to determine whether the patient wants their test results and whether it would be in their best interest to receive these results.

There are also several factors during the act of disclosure that may increase patient understanding and response to learning their result. Providing test results in a binary fashion (i.e., positive or negative based on a predetermined cutoff value) increases the validity and interpretability of results but often leaves patients unsatisfied. Providing quantitative results can be difficult, but comprehension can be increased through the use of specialized communication techniques such as using natural frequencies instead of percentages (e.g., 3 out of 4 vs. 75 %), visual aids, and take-home education materials [48]. Having patients teach back the results and implications of the results is one method of ensuring comprehension. Encouraging the patient to ask questions regularly throughout the visit can also improve comprehension of test results.

Following the disclosure, it is important to follow-up with patients to monitor psychological response and answer any questions that may emerge [49]. Should patients express ongoing negative psychological symptoms in response to learning their results, a behavioral health referral may be valuable. Follow-up calls or visits can also be a great opportunity to counsel patients on risk reduction strategies such as physical activity, sleep hygiene, or nutritional counseling. Finally, post-disclosure follow-up allows for ongoing monitoring of patient’s follow through with treatment recommendations, referrals to specialists, and potential barriers to receiving interventions.

## 6 Resources needed for effective BBM results disclosure in primary care settings

While there are numerous challenges to the implementation of BBM testing and results disclosure in primary care settings, there are some clear resources that could be provided to maximize the effectiveness of this practice. First and perhaps most importantly, clinician training should be prioritized. Specialized trainings focused on interpretation and communication of AD BBMs should be a prerequisite to ordering AD BBMs for PCPs without significant experience in AD and dementia.

Further, materials should be developed to assist PCPs in facilitating results disclosure conversations. These materials could include print or electronic resources that outline key points to address during a disclosure conversation, as well as a frequently asked questions section which provides responses to common questions patients may have about their results. Materials directed towards patients may also be valuable and could include patient educational materials (e.g., describing the pathologies associated with AD and what the specific BBMs ordered are detecting) and decision support materials to aid patients in deciding whether or not to undergo BBM testing. Such decision support materials have been developed by the AGREEDementia group that are specifically targeted to patients with MCI (see https://www.agreedementia.org/education/).

Finding the time in a busy primary care clinic schedule to effectively educate and communicate AD BBM results to patients can be challenging. Given the importance of this topic, consideration of implementing a primary care behavioral health (PCBH) model in clinics serving large populations of older adults may be warranted. The PCBH model emphasizes a team-based approach to managing biopsychosocial issues that present in primary care, with the integration of behavioral health providers providing services in the primary care clinic setting [50]. This approach has been effective in improving psychological symptoms and promoting positive health behavior changes in difficult to manage conditions (e.g., smoking, obesity) [50]. Such an approach could allow for behavioral health specialists, who could receive in depth training in BBM results disclosure, to provide the additional counseling, education, and support necessary to ensure safe and effective results disclosure. This approach is not without its challenges, however, as staffing and resource demands for this model is high and would likely exclude lower resourced practices, thus potentially further exacerbating disparities.

Given that the vast majority of BBM validation studies have been conducted in non-Hispanic White populations who are relatively healthy, interpreting BBM results in diverse patient populations, especially those with medical comorbidities will be challenging. Medical conditions with a high degree of racial disparity, such as chronic kidney disease and obesity, are known to have an effect on BBM levels that is independent of underlying amyloid status [45]. There is an urgent need, prior to clinical implementation, to study the performance of BBMs in diverse populations to ensure that clinical interpretation of results is accurate for all patients.

To achieve widespread implementation of AD biomarkers within primary care settings, several key factors must be addressed. Primary care providers often report feeling inadequately prepared to manage the needs of patients with dementia, particularly in the absence of robust community support [[51], [52], [53]]. This challenge is even more pronounced in rural areas, where access to specialized diagnostic tools and resources is limited [54]. Therefore, establishing networks of specialists and community resources is critically necessary to facilitate the uptake of BBMs.

At the health system level, it will be essential to have clear guidelines pertaining to next steps for patients with a positive BBM test. Implementation of AD immunotherapies has been slow and inconsistent to date; it will be pivotal for health systems to have a defined process in place for workup and referral for such therapies following a positive BBM test. Such guidelines should include how to place a referral for further workup and confirmatory testing, who the dementia specialists in the network are and how to best communicate with them, and what steps that patient will need to take prior to initiation of treatment. In addition to guidelines outlining these processes, special attention should be paid to making the pathways to specialized care and ancillary services as efficient as possible. Providing care navigators to help patients and their loved ones through this process is critical given the complexities of the current landscape. Disclosure of BBM results with no actionable next steps for patients is, at best, confusing, and at worst, potentially harmful. Additionally, the potential risk of overwhelming healthcare systems with increased demand must be carefully considered before implementing blood-based biomarkers [55]. Clear guidelines and protocols need to be established for who is eligible for testing to avoid unnecessary strain on the system. Robust technological infrastructure is needed to support the integration of biomarker data into electronic health records and to facilitate access to specialists through telemedicine, especially in underserved areas [56]. Furthermore, clear insurance and reimbursement policies must be established to ensure that biomarker testing and subsequent treatments are accessible and affordable for all patients. Collaborative care models should be developed to ensure a coordinated and effective approach to managing AD, enhancing both provider preparedness and patient outcomes [57].

## 7 Summary

The landscape of AD diagnosis and treatment has been transformed in recent years with the advent of novel immunotherapies and the development of BBMs of AD pathology. With these shifts comes an urgent need for efficient and accessible testing of patients with cognitive decline. Existing practices which rely on specialized memory clinics will not be able to meet the demands likely to follow from increased awareness of AD and the availability of disease modifying therapies. The primary care clinic is the ideal setting for AD screening using BBMs in the community. PCPs have long been critical in screening for numerous diseases and offer several advantages for BBM testing including a comprehensive understanding of their patient’s health and opportunities for more prompt assessment following onset of cognitive symptoms, potentially reducing delays in obtaining care. However, there are substantial challenges regarding the practice of interpreting and disclosing BBMs in this setting that must first be overcome. Drawing from the accumulated knowledge regarding genetic risk and biomarker test disclosure in AD can provide some guidance for health systems to adjust workflows to maximize patient-centered care in AD screening. Yet, there is little research on disclosure practices for BBMs and in clinical settings as opposed to research, thus further research in these areas is critical.

Appropriate use recommendations for BBMs in AD have been previously published [58]. These guidelines strongly cautioned against using BBMs in primary care settings at that time, noting that additional data is needed. Several ongoing studies (e.g., SUNBIRD, BioFinder-PC) [59] will provide this data in the near future. As this data becomes available and our understanding of the accuracy of BBMs in primary care settings increases, we strongly advise revised appropriate use recommendations focused on the use of BBMs in primary care, with special attention paid to disclosure practices, as a necessary prerequisite prior to implementation.

## Funding

Corey Bolton is funded through support from the Alzheimer's Association (AACSF-22–924002; PI: Bolton) and the National Institute on Aging (K23-AG084850; PI: Bolton). Nicole R. Fowler is funded through support from the National Institute on Aging NIA R01 AG056315, R01 AG059613; PI: Fowler) and the Department of Defense (#W81XWH-17; PI: Fowler). Annalise Rahman-Filipiak is funded through support from the National Institute on Aging (NIA 1K23AG07004401-A1; PI: Rahman-Filipiak). Judith Heidebrink is supported by funding from the National Institutes of Health (P30-AG072931).

## Declaration of generative AI and AI-assisted technologies in the writing process

Neither generative AI nor AI-assisted technologies were used in the writing process for this manuscript.

## CRediT authorship contribution statement

**Corey J. Bolton:** Writing – review & editing, Writing – original draft, Supervision, Project administration, Funding acquisition, Conceptualization. **Ayda Rostamzadeh:** Writing – review & editing, Writing – original draft. **Nathaniel Chin:** Writing – review & editing, Writing – original draft. **Nicole R. Fowler:** Writing – review & editing, Writing – original draft. **Judith Heidebrink:** Writing – review & editing, Writing – original draft. **Annalise Rahman-Fillipiak:** Writing – review & editing, Writing – original draft. **Raymond R. Romano:** Writing – review & editing, Writing – original draft. **Lindsay R. Clark:** Writing – review & editing, Writing – original draft, Conceptualization.

## Declaration of competing interest

The authors declare the following financial interests/personal relationships which may be considered as potential competing interests:

Corey Bolton reports financial support was provided by National Institute on Aging. Corey Bolton reports financial support was provided by Alzheimer’s Association. Nicole Fowler reports financial support was provided by National Institute on Aging. Nicole Fowler reports financial support was provided by US Department of Defense. Annalise Rahman-Filipiak reports financial support was provided by National Institute on Aging. Judith Heidebrink reports financial support was provided by National Institute on Aging. If there are other authors, they declare that they have no known competing financial interests or personal relationships that could have appeared to influence the work reported in this paper.
